# Flavonolignans inhibit the arachidonic acid pathway in blood platelets

**DOI:** 10.1186/s12906-017-1897-7

**Published:** 2017-08-10

**Authors:** Michal Bijak, Joanna Saluk-Bijak

**Affiliations:** Department of General Biochemistry, Faculty of Biology and Environmental Protection, University of Lodz, Pomorska 141/143, 90-236 Lodz, Poland

**Keywords:** Flavonolignans, Silybin, Silychristin, Silymarin, Arachidonic acid, Blood platelet, Cyclooxygenase

## Abstract

**Background:**

Arachidonic acid metabolism by cyclooxygenase (COX) is a major pathway for blood platelets’ activation, which is associated with pro-thrombotic platelet activity and the production of pro-inflammatory mediators. Inhibition of COX activity is one of the major means of anti-platelet pharmacotherapy preventing arterial thrombosis and reducing the incidence of cardiovascular events. Recent studies have presented that a silymarin (standardized extract of Milk thistle (*Silybum marianum*)) can inhibit the COX pathway. Accordingly, the aim of our study was to determine the effects of three major flavonolignans (silybin, silychristin and silydianin) on COX pathway activity in blood platelets.

**Methods:**

We determined the effect of flavonolignans on arachidonic acid induced blood platelet aggregation, COX pathway metabolites formation, as well as COX activity in platelets. Additionally, we analysed the potential mechanism of this interaction using the bioinformatic ligand docking method.

**Results:**

We observed that tested compounds decrease the platelet aggregation level, both thromboxane A_2_ and malondialdehyde formation, as well as inhibit the COX activity. The strongest effect was observed for silychristin and silybin. In our in silico study we showed that silychristin and silybin have conformations which interact with the active COX site as competitive inhibitors, blocking the possibility of substrate binding.

**Conclusions:**

The results obtained from this study clearly present the potential of flavonolignans as novel antiplatelet and anti-inflammatory agents.

## Background

Blood platelets are the smallest, un-nucleated morphotic elements of human blood that play a major role in the blood coagulation system. The biological activity of platelets, both in physiological processes as well as under pathological conditions, is dependent on the degree of their activation. A platelet’s activation process, despite the absence of a nucleus, is very complex and associated with elements of enzymatic signal transduction chains [1]. After the platelets’ activation, signal transduction leads to mobilization of intracellular calcium ions (Ca^2+^). High intracellular concentration of Ca^2+^ results in activation of phospholipases, which are responsible for the release of cell membrane phospholipids’ enzymatic hydrolyses. These include, for example, the precursor of essential bioactive eicosanoids – 5,8,11,14-eicosatetraenoic acid called arachidonic acid (AA), which is a 20-carbon polyunsaturated fatty acid. AA released from the membranes is enzymatically oxidized, transformed by the cyclic peroxide prostaglandin synthase known as cyclooxygenase (COX) into intermediate products: pro-inflammatory prostaglandins and pro-thrombotic thromboxane A_2_ (TXA_2_) [2, 3]. These are accompanied by production of reactive oxygen species (ROS) [4]. TXA_2_ is generated from prostaglandin H2, formed by COX through thromboxane-A synthase. TXA_2_ is an autocrine or paracrine mediator in the nearby tissues surrounding its production site. TXA_2_ is a very strong blood platelet activator acting as a pro-aggregator and vasoconstrictor mediator, leading to increased platelet aggregation. This plays a pivotal role in the growth and stabilization of a coronary thrombus [5]. TXA_2_ is formed in platelets in response to local stimuli and exerts an activating effect within a short distance of its biosynthesis.

AA metabolism by COX is a major pathway of blood platelets activation, and is associated with pro-thrombotic platelets’ activity and the production of pro-inflammatory mediators. AA addition in vitro to platelet rich plasma causes a burst of oxygen consumption, TXA_2_ generation and platelet aggregation [6].

One of the major method in anti-platelet pharmacotherapy of preventing arterial thrombosis is inhibition of COX activity. The results of clinical studies have shown that intake of aspirin, or different aspirin-like COX-inhibitors, reduces the incidence of cardiovascular events [7]. Low-dose aspirin (40 mg per day) supplementation reduces the risk of serious cardiovascular events by 12% and non-fatal myocardial infarction by 18%. This dose is able to inhibit a large proportion of thromboxane A_2_ release provoked acutely by the platelets’ response. Aspirin is also able to reduce the risk of secondary thrombotic events by about 25% [8].

Experiments performed on human monocytes have shown that flavonolignans – active chemical compounds presented in a silymarin (standardized extract from of Milk thistle (*Silybum marianum*)) inhibit the COX pathway [9]. In accordance with this observation, the aim of our study was to determine the effects of three major flavonolignans (silybin, silychristin and silydianin) on COX pathway activity in blood platelets.

## Methods

### Reagents

Dimethyl sulfoxide (DMSO), 3-[(3-Cholamidopropyl)dimethylammonio]-1-propanesulfonate (CHAPS), 4-(2-Hydroxyethyl)piperazine-1-ethanesulfonic acid (HEPES), glucose, trichloroacetic acid, thiobarbituric acid, Tris, flavonolignans (silybin, silychristin and silydianin) were all obtained from the Sigma-Aldrich Chemical Co. (St. Louis, MO, USA). Arachidonic acid was purchased from Chrono-Log (Havertown, PA USA). All other chemicals were reagent grade or the highest-quality available.

### Blood samples

Blood samples collected from different healthy donors were purchased from the Regional Center for Transfusion Medicine in Lodz (Poland). All samples were drawn in the morning, from fasting donors. All donors were checked by a medical doctor and found to have no cardiovascular disorders, allergy, lipid or carbohydrate metabolism disorders, nor were they being treated with any drugs [10]. Our analysis of the blood samples was performed under the guidelines of the Helsinki Declaration for Human Research, and approved by the Committee on the Ethics of Research in Human Experimentation at the University of Lodz (Resolution No. 16/KBBN-UŁ/II/2016).

### Isolation of platelet-rich-plasma and blood platelets

The blood was centrifuged (200×*g*, 10 min, RT) to isolate the platelet rich plasma (PRP). The obtained PRP was then used to measure aggregation and platelet isolation. Blood platelets were isolated by differential centrifugation of blood, as described above [11, 12]. The final concentration of platelet suspension was about 4 × 10^8^ platelets/ml. The platelets were counted using a photometric method according to Walkowiak et al. [13]. The platelet washing procedure was performed in plastic tubes and carried out at room temperature. Washed human platelets were suspended in a modified Tyrode’s Ca^2+^/Mg^2+^ free buffer (127 mM NaCl, 2.7 mM KCl, 0.5 mM NaH_2_PO_4_, 12 mM NaHCO_3_, 5 mM HEPES, 5.6 mM glucose, pH 7.4).

The isolated platelets, as well as the PRP samples, were pre-incubated with flavonolignans (silybin, silychristin and silydianin) in a concentration range of 10–100 μM by 30 min at 37 °C. All tested compounds were initially dissolved in 20% DMSO to a preliminary concentration of 20 mM. Other solutions of the compounds used were also performed in 20% DMSO (prepared in 50 mM TBS, pH 7.4). The final DMSO concentration of all samples was 0.1%. In control samples the same volume of solvent was added (20% DMSO prepared in 50 mM TBS, pH 7.4), with the probes warmed for 30 min at 37 °C. The isolated and purified blood platelets were used to determine the level of TBARS and activity of COX. Samples dedicated to the determination of COX activity were dissolved in 1:1 Cell Lysis Buffer (BD Pharmingen™).

### Platelet aggregation induced by AA

Platelet aggregation was measured turbidimetrically in PRP using the optical Chrono-Log aggregometer (Chrono-Log, Havertown, PA). The PRP samples were pre-incubated with flavonolignans (silybin, silychristin and silydianin) at the concentration range of 10–100 μM by 30 min at 37 °C. All tested compounds were initially dissolved in 20% DMSO (prepared in 50 mM TBS, pH 7.4) to the preliminary concentration of 20 mM. The final concentration of DMSO in all samples was 0.1%. In the control samples, the same volume of solvent was added (20% DMSO prepared in 50 mM TBS, pH 7.4), with the probes warmed for 30 min at 37 °C. The prepared PRP samples were pre-warmed at 37 °C and stirred. After the pre-incubation procedure for the PRP samples, the free AA (1 mM) were added and platelet aggregation measured for 10 min. The aggregometer was calibrated each time (100% aggregation) on platelet poor plasma (PPP), with the appropriate concentration of each flavonolignan.

### Determination of COX-1 activity

The level of COX-1 activity in the platelet lysates was determined by the fluorescence-based method, using a COX Fluorescent Activity Assay Kit (Cayman Chemicals). The oxygenase activity of COX causes conversion of the arachidonic acid to a prostaglandin G2 (PGG_2_) which is the first intermediate in the COX pathway. This assay is based on the reaction formed between PGG_2_ and 10-acetyl-3,7-dihydroxyphenoxazine (ADHP), which produces the highly fluorescent compound Resorufin. This can be analysed using an excitation wavelength of 530–540 nm and an emission wavelength of 585–595 nm. The activity of COX is presented herein as the amount of Resorufin produced per time unit [nmol/min]. Each step of procedure was performed according to manufacturer protocol.

### Estimation of thiobarbituric acid reactive substances

Samples of blood platelet suspended in the modified Tyrode’s buffer were treated with thrombin (1 U/ml). After 10 min the samples were mixed with an equal volume of 15% (*w*/*v*) cold trichloroacetic acid, and with an equal volume of 0.37% (*w*/*v*) thiobarbituric acid in 0.25 M HCl. All samples were immersed in a boiling water bath for 10 min. After cooling, the samples were centrifuged and then absorbance was measured at 535 nm. The results were estimated using a molar extinction coefficient of malondialdehyde (MDA), a reliable marker of lipid peroxidation (ε = 1.56 × 10^5^ M^−1^ cm^−1^), and expressed as a percent of control value, as described previously [14].

### Determination of the level of TXB_2_ using a competitive ELISA assay

All of the blood samples were pre-incubated with flavonolignans (silybin, silychristin and silydianin), in a concentration range of 10–100 μM for 30 min at 37 °C. In the control samples, the same volume of stock flavonolignans solvent was added (20% DMSO prepared in 50 mM TBS, pH 7.4), with the probes warmed for 30 min at 37 °C. After that, the samples were transferred into Sarstedt S-Monovette Serum tubes with a coagulation activator (thrombin 2.5 U/ml), and left at 37 °C. After 30 min the samples were centrifuged (4500 rpm, 15 min, 37 °C) to obtain serum. To quantify the level of Thromboxane B2 (TXB_2_) in the samples (the serum was diluted 100 times), a Thromboxane B_2_ Express ELISA Kit – Monoclonal (Cayman Chemicals) was used. The total level of TXB_2_ in all samples was obtained as ng/ml using a calibration curve and expressed as a percent of control value.

### Ligand docking

The ligand dockings for the selected flavonolignans to the cyclooxygenase were calculated in silico with Autodock Vina 1.0, an algorithm released by Scripps Research Institute [15] (http://vina.scripps.edu/), in accordance with the previously used procedure [16–18]. The protein coordinates and PDB format structure of the cyclooxygenase-1 3N8Z [19] were taken from the website of the RSCB Protein Data Bank (http://www.rcsb.org). All three-dimensional chemical structures of the flavonolignans were downloaded from PubChem (http://pubchem.ncbi.nlm.nih.gov/) and converted to.pdb files using Avogadro 1.1.0, an open-source molecular builder and visualization tool (http://avogadro.openmolecules.net/) [20]. Protein preparation for the docking procedure was performed in a Swiss-PdbViewer (http://spdbv.vital-it.ch/). The non-bonded atoms present in the crystal structure were removed. Next, the receptor structure was adapted in Auto Dock Tools v 1.5.6rc1 (http://autodock.scripps.edu) [21] and the missing hydrogen atoms, Gasteiger charges [22] as well as Kollman [23] united atom charges were calculated and assigned. Non-polar hydrogens were merged, and rotatable bonds assigned, keeping all the amide bonds as non-rotatable. The protein file was prepared in.pdbqt format, which is pdb plus “q” charges and the “t” AutoDock type. The docking procedure was run with the following settings: center_x − 21.039; center_y 53.126; center_z 10.252; size_x 126; size_y 126; size_z 126. The binding points were computed and the binding affinity of the ligand to the receptor counted in kcal/mol. Analysis and visualization of the three-dimensional structures of the protein with the bound ligand was performed with the Python Molecular Viewer of Auto Dock Tools v 1.5.6rc1 (http://autodock.scripps.edu) [21], and Swiss-PdbViewer (http://spdbv.vital-it.ch/) [24]. The docking protocol procedure was validated using flurbiprofen compound, which was docked to the same place as presented in the crystal structure. This indicated the reliability of the presented docking method.

### Data analysis

The statistical analysis was performed using StatsDirect statistical software V. 2.7.2. All experimental values presented in this study were expressed as mean ± standard deviation (SD). To analyse the normality of the distribution of results, the Shapiro-Wilk test was used. Next, the results were analysed under the equality of variance using Levene’s test. The significance of the differences between the values was analysed using ANOVA, followed by Tukey’s range test for multiple comparisons (for data with normal distribution and equality of variance), and the Kruskal-Wallis test; *p* < 0.05 was accepted as statistically significant [25–28].

## Results

**Fig. 1:**
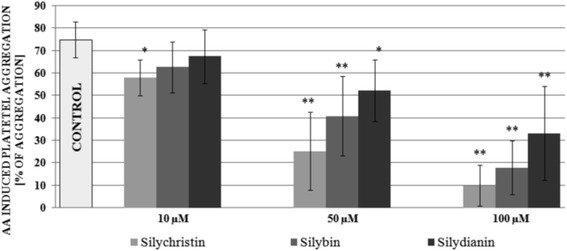
The effect of flavonolignans (silychristin, silybin and silydianin in concentrations of 10; 50 and 100 μM) on blood platelet aggregation induced by arachidonic acid in platelet-rich plasma. The data represents means of ± SD, *n* = 12. Statistical analysis was performed using Tukey’s Range Test, *p < 0.05, **p < 0.001

In the first step of our study we examined the effect of flavonolignans on COX pathway-induced blood platelets aggregation. To ensure full platelet activation, a physiological stimulus was added – AA (1 mM). In our measurements we found that in a dose-dependent manner, all tested flavonolignans inhibit the activation of platelets in PRP upon AA stimulation, resulting in a statistically significant dose-dependent decrease in the platelet aggregation level. The strongest inhibitory effects were observed for silychristin and silybin, which at the highest used concentration (100 μM) reduced AA induced blood platelets aggregate formation (control value of 75%), to 10% and 18% respectively. In the samples treated with silydianin we also observed an inhibitory effect (Fig. 1). However, it was lower in comparison to the silybin and silychristin.

**Fig. 2:**
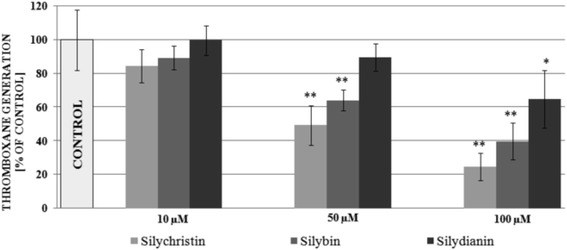
The effect of flavonolignans (silychristin, silybin and silydianin at concentrations 10; 50 and 100 μM) on TXA_2_ generation in blood platelets. The concentration of TXA_2_ metabolite (TXB_2_) was measured in serum obtained after blood coagulation activation by thrombin. The data represents means of ± SD, *n* = 12. Statistical analysis was performed using Tukey’s Range Test *p < 0.05, **p < 0.001

**Fig. 3:**
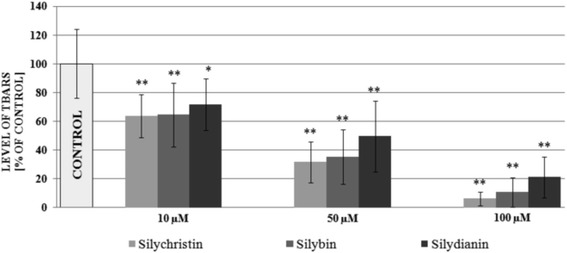
The effect of flavonolignans (silychristin, silybin and silydianin at concentrations 10; 50 and 100 μM) on the blood platelets COX metabolite pathway was estimated by the level of TBARS concentration measurement. The data represents means of ± SD, *n* = 12. Statistical analysis was performed using the Kruskal-Wallis test, *p < 0.05, **p < 0.001

Next, we established the effect of flavonolignans on the generation of the main COX pathway metabolite: thromboxane A2. Generation of TXA_2_ was determined by measurement of the concentration of its stable metabolite TXB_2_, using the ELISA method. In all samples where blood had been treated with flavonolignans, a reduction of TXB_2_ concentration was observed (Fig. 2). In samples treated with silychristin and silybin (100 μM), the levels of generated thromboxane were about 75% and 60% reduced, respectively. In the case of the highest concentration of silydianin, the level of generated thromboxane was reduced by about 35%. Our results also revealed that in a dose-dependent manner, flavonolignans inhibit synthesis of AA metabolites, measured as the amount of TBARS and expressed as nmoles of MDA per ml of platelet suspension (Fig. 3).

**Fig. 4:**
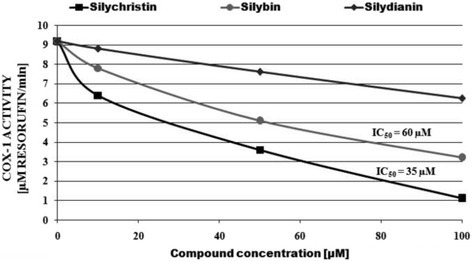
The effect of flavonolignans (silychristin, silybin and silydianin at concentrations 10; 50 and 100 μM) on the level of oxygenase activity of COX-1 in platelets. The Figure shows the average rate of change of Resorufin formation. The data represents the means of 12 measurements

In the next step of our study, we determined the direct effect of flavonolignans on the oxygenase activity of COX-1, measured in blood platelets. We observed that all examined flavonolignans caused the inhibition of COX activity (Fig. 4). COX inhibition by the tested compounds was expressed as a IC_50_ value – the concentration needed to inhibit 50% of enzyme activity. The strongest inhibitory effect was demonstrated by silychristin and silybin (IC_50_ for silychristin was 35 μM, 60 μM for silybin), while for silydianin we did not obtain an IC_50_ value (at the maximum tested concentration of 100 μM, a 32% inhibition of COX activity was observed).

**Fig. 5:**
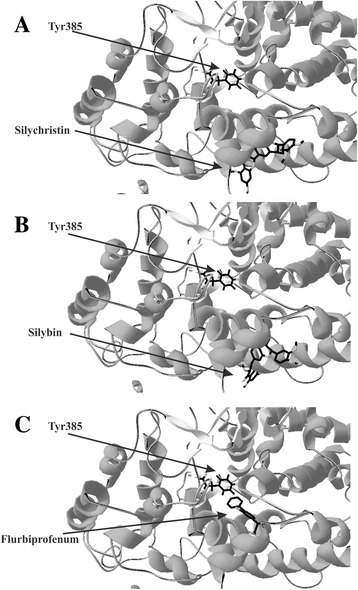
3D structures of ligand docking to the binding site of cyclooxygenase. The crystal structure of cyclooxygenase (PDB: 3N8Z was taken from the RCSB PDB databank (http://www.rcsb.org/). 3D ligands structures were obtained from the PubChem website (http://pubchem.ncbi.nlm.nih.gov/). Software used for docking was Autodock Vina 1.0 together with Autodock Tools v 1.5.6rc1. Visualization of the docking results (conformation with the highest affinity to the active centre), was rendered using Swiss-PdbViewer; A – silychristin, B – silybin, C – flurbiprofenum

In our in silico study we computer-generated models of interaction between COX and selected flavonolignans (silychristin and silybin). We observed that both tested compounds have conformities which interact with active site of COX, blocking the possibility of substrates binding. B rings of Silychristin and silybin (Fig. 5) bind to the entry of active site loop where is located Tyr385 residue. The obtained affinity parameters demonstrate that silychristin and silybin have a strong binding mode to COX active site entry (−9.8 kcal/mol and −9.2 kcal/mol respectively), in comparison to flurbiprofenum, one of the most popular non-steroid anti-inflammatory drugs (−8.9 kcal/mol).

## Discussion

Cyclooxygenase is a representative of membrane-bound glycoproteins and possesses two distinct enzymatic activities: oxygenase and peroxidase. In the early 1990s, identification of two members of the COX family was made. COX-1 is constitutively expressed in many types of cells, however it is the only COX form expressed in blood platelets. COX-2 is an inducible form which is activated in cells by pro-inflammatory inflammatory cytokines [29].

COX-pathway activation in blood platelets is strictly dependent on its substrate – the unesterified free form of arachidonic acid released from the internal phospholipid membrane [30]. AA is the main fatty acid component of phospholipids in blood platelets’ cell layer (phosphatidylcholine, phosphatidylethanolamine and phosphatidylinositol may contain even 80% of AA) [2]. After being released by phospholipase A_2_ and phospholipase C, free AA is oxidized at C-11 by COX. The oxygenase activity of COX catalyses the incorporation of two oxygen atoms to one molecule of arachidonate, creating a prostaglandin G2 (PGG_2_). Next, peroxidase catalysis results in reduction of the 15-OOH group in the PGG_2_ structure to the 15-OH group, and formation of prostaglandin H2 (PGH_2_). PGH_2_ is a substrate for enzymes in the heme prosthetic group – thromboxane A (TXA) synthase. The heme group presented in the catalytic centre of TXA synthase is responsible for homolytic cleavage of the epidioxy bond in PGH_2_, and rearrangement to TXA_2_ [31].

One of the direct actions of thromboxane on blood platelets is to modify the response of platelets to exogenous agonists, as well as to stimulate platelet activation. The platelet’s response to TXA_2_ is responsible for the surface receptor for TXA_2_ (TP), which is a member of the seven-transmembrane G-protein-coupled receptor family. Activation of TP results in both the autocrine and paracrine action of TXA_2_ released from blood platelets. The TP signalling pathway is mediated by two G proteins: G_q_ and G_12/13_. G_q_ is responsible for activation of phospholipase C, resulting in increased inositol 1,4,5-triphosphate concentration. In consequence, intra-cellular Ca^2+^ ions are mobilised and diacylglycerol is formed, which activates protein kinase C. These events cause platelet shape change, aggregation, and secretion from intra-cellular granules. Stimulation of G_12/13_ subunits activates the Rho pathway, which results in myosin light chain phosphorylation and subsequent platelet shape change [32].

TXA_2_ is a very unstable molecule with a short half-life of about 30 s, after which it passes to the very stable but inactive metabolite TXB_2_ [32].

The involvement of blood platelets in both the physiological coagulation process and pathological conditions is dependent on their activation level. The non-stimulated (resting) blood platelets lacked any free AA and therefore blocked the COX. After activation, an intracellular Ca^2+^ flux activated both enzymes: phospholipases C and A_2_, which hydrolyse membrane phospholipids and release free AA [33].

The exogenous arachidonic acid is able to cause irreversible platelet aggregation, as it can be rapidly incorporated into membrane phospholipids, primarily phosphatidylcholine and phosphatidylinositol, by arachidonoyl coenzyme A synthetase. Our current studies have demonstrated that flavonolignans are able to inhibit platelet aggregation induced by extracellular AA. This suggests the ability of the tested flavonolignans to block the arachidonic acid metabolism pathway in blood platelets. Additionally, in probes pre-incubated with flavonolignans we also observed inhibition of the formation of COX pathway metabolites: MDA and thromboxane A_2_. Following this, we determined the direct effect of flavonolignans on COX activity and saw that silychristin and silybin have very strong inhibitory properties with low IC_50_ values (35 μM and 60 μM respectively).

The final step of our study, to explain the potential mechanism of obtained experimental results, was to characterise the interaction between selected flavonolignans and COX structure using bioinformatic ligand docking. In silico docking methods have undergone significant developments and improvements over the last few years [34]. Docking software computes the best conformation and placement of ligands in a receptor structure. For our study, we used the AutoDock Vina programme, which combines some of the advantages of knowledge-based potentials and empirical scoring functions. It extracts empirical information from both the conformational preferences of the receptor-ligand complex and from experimental affinity measurements [15]. The results obtained from the docking demonstrated the potential direct mechanism of interaction between flavonolignans (silychristin and silybin) and the active COX centre. This interaction block entry to the COX loop where is located Tyr385 residue. Tyr385 generates a tyrosyl radical in the cyclooxygenase active site which is abstracts the pro-S hydrogen atom from carbon-13 of AA, initiating the cyclooxygenase reaction [31, 35].

Acetylsalicylic (ASA) acid was the first cyclooxygenase inhibitor to be recognized and introduced to clinical use, and has been shown to be effective in reducing cardiovascular disease conditions associated with thrombosis, and increased blood platelet activation. The molecular mechanism of this inhibition is ASA’s ability to cause irreversible acetylation of the platelet cyclooxygenase, with serine residue of: Ser529 of COX-1 and Ser516 of COX-2. This acetylation results in blocking of the access of arachidonic acid to the active enzyme site, which leads to irreversible inhibition of the formation of both prothrombotic and pro-inflammatory mediators in blood platelets [36, 37]. ASA is the most popular substance in prevention of cardiovascular pathologies and has a well-established role in preventing thrombotic events in patients with recognized cardiovascular disease. Additionally, large-scale clinical studies conducted last years, demonstrated the effectiveness of ASA in the primary prevention of thrombotic events in men and women without diagnosed cardiovascular disease. In these studies, ASA significantly reduced the risk of a first myocardial infarction, stroke or death from cardiovascular causes [38, 39]. However, ASA treatment is subject to risks from bleeding. Meta-analysis performed by Baigent et al. [8] demonstrated that long-term ASA administration increases the risk of major gastrointestinal and other extracranial bleedings by 0.5%. In a study by De Berardis et al. [40], the risk of bleeding increased by 2.5% in the ASA treatment group. ASA also increases the risk of haemorrhagic stroke [41]. These facts illustrate the necessity of ASA therapy to give proven clinical benefits, and the need for its assessment using precise estimation of benefits and risks, especially for rare events, such as intracranial haemorrhage [38]. For these reasons, all over the world incessant research is taking place on novel, therapeutically better, more effective anti-platelet agents that would have a significant effect on proper haemostatic stability, without adverse effects. Plants are a good source for isolating natural compounds without unfavourable side effects, capable of inhibiting the enzymes involved in cell signalling pathways and reducing blood platelets activation [42].

## Conclusions

The results obtained from this study clearly present the potential role of flavonolignans, natural-origin compounds present in fruits of Milk thistle, an *Asteraceae* family plant (*Silybum marianum*, L. Gaernt.), as novel antiplatelet and anti-inflammatory agents. Since the 1970s, these compounds have been used as official medicine and are used in treatment of liver dysfunctions. Furthermore, as yet side effects from flavonolignans have not been found [43]. Additionally, in the last few years, novel forms of administration of Milk thistle extract have been discovered that have very high bioavailability (with plasma concentrations ranging from 60 to 70 μM) [44]. This corresponds with the concentrations of flavonolignans that have a biological effect in our study (10–100 μM).
